# The clinical utility of handgrip strength as a malnutrition screening tool in hospitalized older adults: a cross-sectional study in Saudi Arabia

**DOI:** 10.3389/fmed.2024.1436977

**Published:** 2024-07-23

**Authors:** Sultan H. Alamri, Mayar M. Simbawa

**Affiliations:** ^1^Department of Family Medicine, Faculty of Medicine, King Abdulaziz University, Jeddah, Saudi Arabia; ^2^Neuroscience and Geroscience Research Unit, King Fahd Medical Research Center, Abdulaziz University, Jeddah, Saudi Arabia; ^3^Department of Family Medicine, King Abdulaziz University Hospital, Jeddah, Saudi Arabia

**Keywords:** handgrip strength, nutritional status, older adults, geriatric, Saudi Arabia

## Abstract

**Background:**

Malnutrition is prevalent among hospitalized older patients. Early identification is therefore essential to implementing appropriate therapeutic interventions. This study aimed to explore the correlation between handgrip strength (HGS) and nutritional status in hospitalized older adults.

**Materials and methods:**

This observational cross-sectional study was conducted at King Abdulaziz University Hospital, where a consecutive cohort of older adult inpatients was enrolled for participation. Shortly after admission, HGS and nutritional status were assessed using a dynamometer and the most recent version of the Mini-Nutritional Assessment Short Form (MNA-SF) test, respectively. Key anthropometric and biochemical indicators were also collected.

**Results:**

A total of 135 consecutive patients were evaluated. Among participants with low HGS, 18 (16.22%) were malnourished, 43 (38.74%) were at risk of malnutrition, and 50 (45.05%) had normal nutrition status. The participants with low HGS had low hemoglobin, low lymphocyte levels, high creatinine levels, high BUN levels, high CRP levels, high HbA1c levels, and high vitamin B12 levels. Multiple logistic regression analysis showed that age, hemoglobin, and HbA1C were independently associated with low HGS. Based on the cut-off values for the HGS by the European Working Group on Sarcopenia in Older People-2 (EWGSOP2), low HGS showed high sensitivity to detect “malnourished and at risk of malnutrition” as well as “malnourished alone;” however, the specificity was low to exclude “malnourished and at risk of malnutrition” as well as “malnutrition alone.”

**Conclusion:**

Age over 75 years, low hemoglobin, and elevated HbA1C levels were independent risk factors for low HGS. While HGS was sensitive in detecting malnutrition or risk, its specificity was low. Therefore, HGS may not be adequate as a standalone tool to assess nutritional status in hospitalized older adults. Replication of this study using locally reliable and validated HGS cut-off values is warranted to confirm these findings.

## 1 Introduction

Malnutrition is defined as “a state resulting from the lack of appropriate nutritional intake or uptake that leads to altered body composition (decreased fat-free mass) and body cell mass, leading to diminished physical and mental function and an altered clinical outcome from disease” (1). Nutritional risk or malnutrition is prevalent among hospitalized older patients (2). According to a systematic review, the prevalence rates of individuals at high risk of malnutrition were estimated to be 28.0% in hospital settings and 8.5% in community settings (2). In the hospital setting, malnutrition has been independently linked to a higher risk of adverse consequences, including longer hospital stays, more complications, increased mortality, and higher healthcare expenditures (3, 4). Malnourished patients are also more susceptible to infections, impaired wound healing, and reduced muscle function, all of which contribute to a reduced quality of life and an overall poor prognosis (4). Early identification is therefore essential to mitigate these negative impacts and improve clinical outcomes (5). Traditionally, nutritional status assessment has relied on biochemical markers to identify malnourished patients. However, functional assessment, HGS in particular, has recently gained considerable attention as a potential indicator of nutritional status, signifying a shift in the approach to nutritional assessment (6, 7).

Although a definitive conclusion on the utility of HGS is lacking, research on its correlation with nutritional status is currently limited across many countries, including Saudi Arabia. Hence, the present study aimed to explore whether HGS is a predictor of nutritional status in a cohort of hospitalized older adults in Saudi Arabia.

## 2 Materials and methods

### 2.1 Study design

This observational cross-sectional study was conducted during April and May 2022 at King Abdulaziz University Hospital, Jeddah, Saudi Arabia. The study protocol was approved by the Research Ethics Committee (REC) of KAUH (Reference No. 591–21), and all participants provided verbal informed consent.

### 2.2 Participants

A total of 135 consecutive patients aged 60 years or older were recruited from the medical and surgical wards of King Abdulaziz University Hospital. Data were obtained directly from participants. Patients who were readmitted during the study period, experiencing terminal illness or upper limb disabilities (neurological or musculoskeletal), or were unable to provide consent were excluded from the study.

### 2.3 Anthropometric and clinical data

Shortly after hospital admission, anthropometric measurements including body mass index (BMI), weight, and height were obtained using standard techniques. Biochemical data were extracted from the electronic medical records including albumin (ALB), total protein (TP), creatinine (Cr), triglycerides (TG), total cholesterol (TC), hemoglobin (Hb), white blood cells (WBC), lymphocytes, blood urea nitrogen (BUN), hemoglobin A1C (HbA1C), and C-reactive protein (CRP). Other information was also collected, such as age, sex, and admission diagnosis.

### 2.4 Nutritional status measurement

The nutritional status of the patients was estimated using the Mini Nutrition Assessment Short Form (MNA-SF). The form was initially developed in 1994 to assess nutritional and functional status among older patients, as well as to predict mortality. It has been widely used, both in research and in clinical practice, as a simple and inexpensive tool with 96% sensitivity and 98% specificity (8). With the revised MNA-SF, quick screening of potential candidates is allowed, and the tool has increased clinical applicability in clinical practice (8, 9). MNA-SF score ranges from 0 to 14. Subjects who score less than 8 are considered malnourished. At-risk participants score between 8 and 11, while those who are well-nourished score above 12.

### 2.5 Handgrip strength measurement

The HGS was measured using an electronic handgrip dynamometer (CAMRY, Model 1 EH101). Before the assessment, a healthcare provider provided verbal instructions and demonstrated the test procedure to each participant. The patients were clearly instructed to squeeze the device firmly for 3 s with their dominant hand. The arm and dynamometer were held on the side of the body with the elbow flexed at 90° (10). Every patient was required to perform three maximal isometric contractions while upright, with a minimum of 10 s and a maximum of 30 s of rest between tests. The highest score was then recorded.

Based on the revised algorithm for the diagnosis of sarcopenia of the European Working Group on Sarcopenia in Older People-2 (EWGSOP2), the low grip strength is defined as <16 kg for women and < 27 kg for men (11).

### 2.6 Statistical analysis

Descriptive statistics were presented for sociodemographic data. The continuous data were presented as mean and standard deviation (SD), and the categorical data were presented as frequencies and percentages. The groups were compared using the Welch two-sample *t*-test for continuous data, and Pearson’s chi-squared test or Fisher’s exact was used for categorical variables as appropriate. Receiver operating characteristic (ROC) analysis is a powerful tool for evaluating the performance of diagnostic tests that deal with binary outcomes. The area under the ROC curve (AUC) is a single numerical value summarizing the overall performance of the model. An AUC of 1 represents perfect discrimination, while an AUC of 0.5 indicates no better than chance performance. ROC analysis was performed to evaluate the predictive performance of HGS (kg) to malnourished and/or at risk of malnourished versus normal and to detect malnourished versus rest (at risk of malnourished and normal). The sensitivity, specificity, and AUC were evaluated for each of the above ROC analyses and provided 95% confidence intervals.

Logistic regression analysis was performed to identify the factors associated with the low HGS. The baseline characteristics and biochemical markers that were significant at group comparisons were used as exposure variables. Stepwise variable selection was adopted to identify the independent variables associated with low HGS. A *p*-value of <0.05 was considered significant. R programming language version 3.6.3 was used for the analysis.

## 3 Results

### 3.1 Baseline characteristics

In this study, 135 participants aged 60 years and older were included; of whom, 50.4% were women (*n* = 68). Approximately one-third (30.4%) of the participants were illiterate, and the majority (68.9%) were married. Approximately three-quarters (73.3%) of the participants were non-smokers, and 38.5% (*n* = 52) had a BMI between 25.0 and 29.9. Diabetes, hypertension, heart disease, cancer, and polypharmacy were the most prevalent conditions among the participants. The baseline characteristics of the study population are presented in Table 1.

**Table 1:** Baseline characteristics of the study participants.

Variable		Number	Frequency (%)
Age group	<75	99	73.3%
	> = 75	36	26.7%
Gender	Women	68	50.4%
	Men	67	49.6%
Occupation	Employed	35	25.9%
	Unemployed or retired	100	74.1%
Educational level	Illiterate	41	30.4%
	Primary school	23	17%
	Middle school	17	12.6%
	Secondary school	32	23.7%
	Higher education	22	16.3%
Marital status	Married	93	68.9%
	Single	7	5.2%
	Widowed	28	20.7%
	Divorced	7	5.2%
BMI	Underweight	6	4.4%
	Healthy weight	36	26.7%
	Overweight	52	38.5%
	Obesity	41	30.4%
Smoking	Yes	36	26.7%
	No	99	73.3%
Diabetes Mellitus	Yes	94	69.6%
	No	41	30.4%
Hypertension	Yes	97	71.9%
	No	38	28.1%
Dyslipidemia	Yes	53	39.3%
	No	82	60.7%
Cardiac disease	Yes	59	43.7%
	No	76	56.3%
Cerebrovascular disease	Yes	29	21.5%
	No	106	78.5%
Kidney disease	Yes	27	20%
	No	108	80%
Osteoarthritis	Yes	14	10.4%
	No	121	89.6%
Depression	Yes	6	4.4%
	No	129	95.6%
Falls	Yes	7	5.2%
	No	128	94.8%
Cancer	Yes	38	28.1%
	No	97	71.9%
Others	Yes	84	62.2%
	No	51	37.8%
Polypharmacy (> = 5 medications)	Yes	55	40.7%
	No	80	59.3%

BMI, body mass index.

### 3.2 Nutritional status assessment

**Figure 1:**
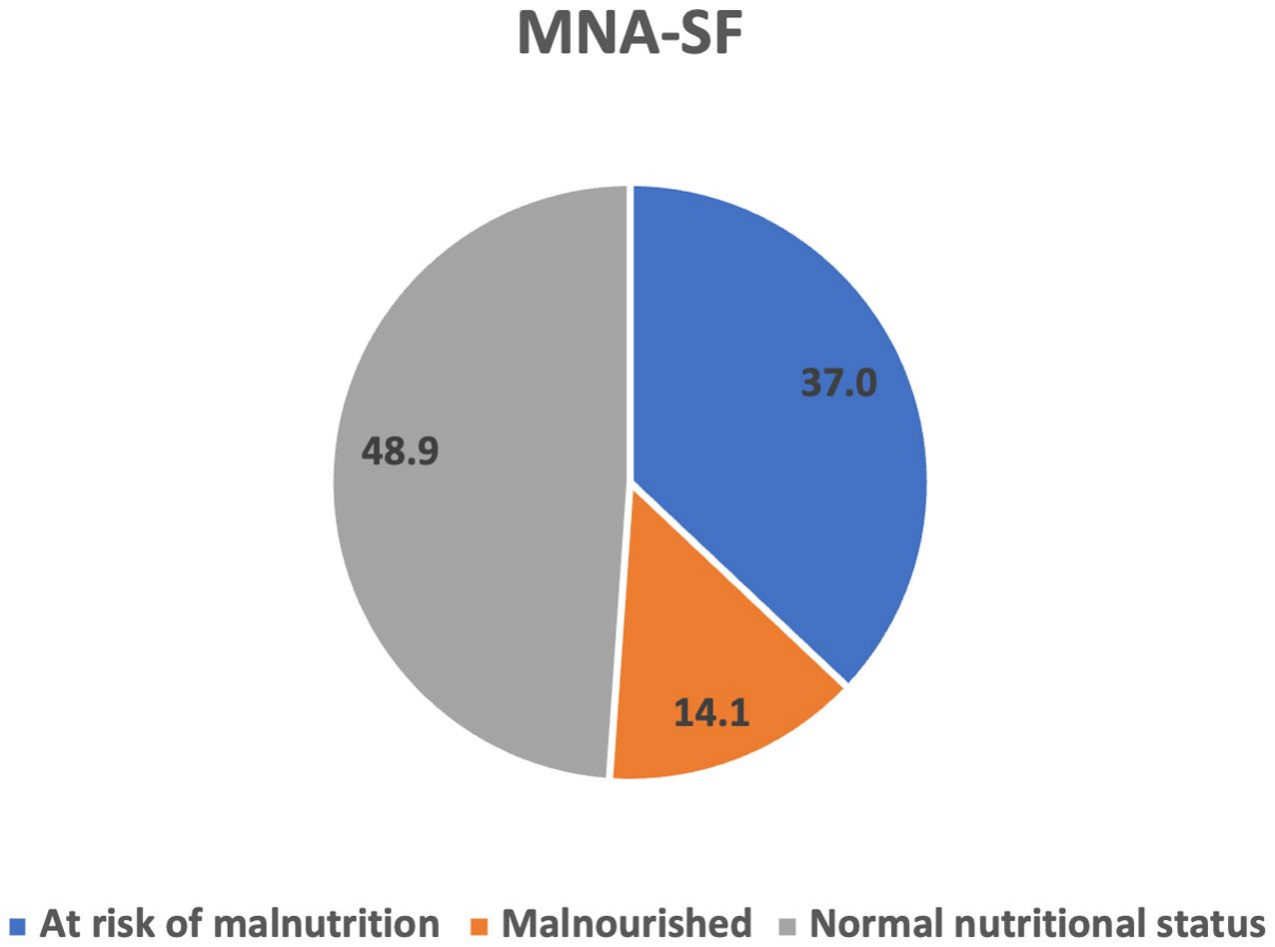
Nutritional status according to MNA-SF.

According to the MNA-SF classification, 14.1% (*n* = 19) of the study participants were malnourished, 37% (*n* = 50) were at risk of malnutrition, and 48.9% (*n* = 66) had normal nutritional status (Figure 1). The MNA-SF score did not differ significantly between men and women.

### 3.3 Handgrip strength assessment

**Figure 2:**
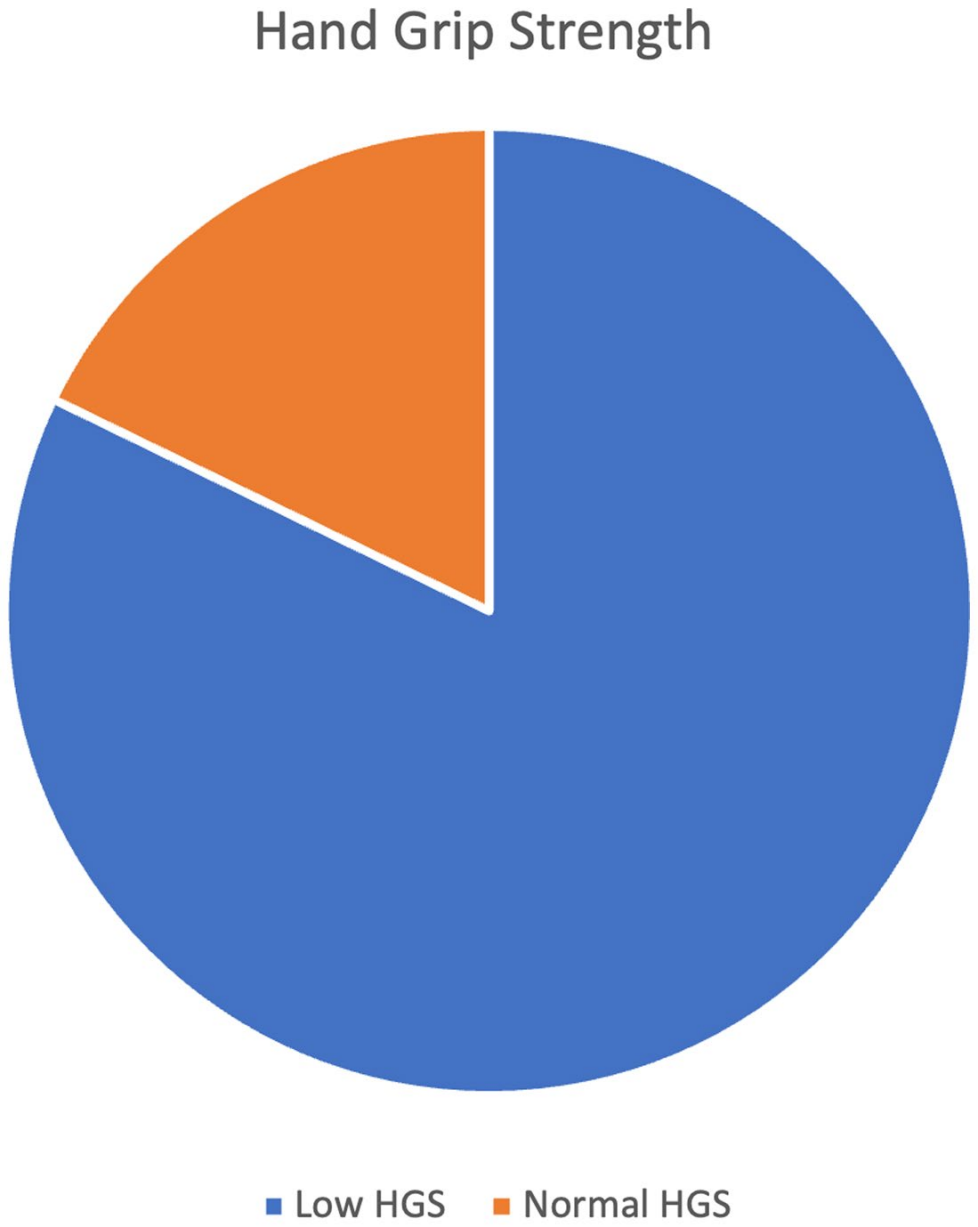
Hand grip strength (HGS).

The average value of the strength of the handgrip was 17.027 kg in men and 10.625 kg in women. Low HGS was prevalent in 82.2% of the participants (Figure 2). There was a statistically significant difference in low HGS with respect to age group, occupation, and education level (*p* < 0.05). A higher percentage of those with low HGS had cardiac diseases compared to those with normal HGS (54 (48.6%) versus 5 (20.8%), *p* = 0.013). Similarly, a higher percentage of those with low HGS had kidney disease compared to those with normal HGS [26 (23.4%) versus 1 (4.16%), *p* = 0.045]. Among those who had normal HGS, 1 (4.16%) was malnourished, 7 (29.16%) were at risk of malnourished, and 16 (66.67%) had normal nutritional status. On the other hand, among those who had low HGS, 18 (16.22%) were malnourished, 43 (38.74%) were at risk of malnutrition, and 50 (45.05%) had normal nutrition status (Table 2).

**Table 2:** The correlation between HGS and baseline characteristics.

Characteristic	Low HGS defined, *N* = 111^a^	Normal HGS defined, *N* = 24^a^	*p*-value^b^
Age group			0.006
<75 years	76 (68%)	23 (96%)	
> = 75 years	35 (32%)	1 (4.2%)	
Gender			0.68
Women	55 (50%)	13 (54%)	
Men	56 (50%)	11 (46%)	
BMI			0.38
Mean (SD)	27.7 (6.0)	28.7 (5.0)	
Occupation			0.014
Employed	24 (22%)	11 (46%)	
Unemployed	87 (78%)	13 (54%)	
Educational level			0.002
Illiterate	38 (34%)	3 (13%)	
Middle school	13 (12%)	4 (17%)	
Primary school	22 (20%)	1 (4.2%)	
Secondary school	26 (23%)	6 (25%)	
Tertiary education	12 (11%)	10 (42%)	
Marital status			0.33
Divorced	4 (3.6%)	3 (13%)	
Married	77 (69%)	16 (67%)	
Single	6 (5.4%)	1 (4.2%)	
Widowed	24 (22%)	4 (17%)	
Smoker	27 (24%)	9 (38%)	0.19
Diabetes mellitus	78 (70%)	16 (67%)	0.73
Hypertension	82 (74%)	15 (63%)	0.26
Dyslipidemia	40 (36%)	13 (54%)	0.10
Cardiac disease	54 (49%)	5 (21%)	0.013
Cerebrovascular disease	24 (22%)	5 (21%)	0.93
Kidney disease	26 (23%)	1 (4.2%)	0.045
Osteoarthritis	11 (9.9%)	3 (13%)	0.71
Depression	6 (5.4%)	0 (0%)	0.59
Falls	4 (3.6%)	3 (13%)	0.11
Cancer	30 (27%)	8 (33%)	0.53
Others	68 (61%)	16 (67%)	0.62
Polypharmacy	49 (44%)	6 (25%)	0.083
MNA-SF Malnourished	18 (16.2%)	1 (4.2%)	0.110
MNA-SF at risk of malnutrition	43 (38.7%)	7 (29.2%)	
MNA-SF normal nutritional status	50 (45.0%)	16 (66.7%)	

a *n* (%).

b Pearson’s Chi-squared test; Welch two-sample *t*-test; Fisher’s exact test.

In addition, the participants with low HGS had low hemoglobin levels (*p* < 0.001), low lymphocyte levels (*p* = 0.040), high creatinine levels (*p* = 0.004), high BUN levels (*p* = 0.007), high CRP levels (*p* = 0.007), high HbA1c levels (*p =* 0.029), and high vitamin B12 levels (*p =* 0.042) (Table 3).

**Table 3:** The correlation between HGS and biochemical markers.

Characteristic	Low HGS defined, *N* = 111	Normal HGS defined, *N* = 24	*p*-value^a^
Hemoglobin			0.001
Mean (SD)	10.34 (2.03)	12.70 (2.06)	
WBC			0.24
Mean (SD)	9.4 (4.1)	8.6 (2.7)	
Lymphocytes			0.040
Mean (SD)	1.83 (0.94)	2.33 (1.06)	
Creatinine			0.004
Mean (SD)	122 (125)	82 (31)	
BUN			0.007
Mean (SD)	9 (7)	6 (4)	
CRP			0.007
Mean (SD)	31 (58)	8 (31)	
HbA1c			0.029
Mean (SD)	7.35 (2.61)	5.83 (3.02)	
Total cholesterol			0.74
Mean (SD)	3.24 (2.00)	3.07 (2.38)	
Triglyceride			0.70
Mean (SD)	1.32 (0.98)	1.22 (1.15)	
Total protein			0.60
Mean (SD)	70 (9)	70 (7)	
Albumin			0.093
Mean (SD)	36.7 (6.7)	39.0 (5.8)	
Vitamin D			0.60
Mean (SD)	39 (35)	33 (52)	
Vitamin B12			0.042
Mean (SD)	297 (327)	181 (224)	

a Welch Two Sample *t*-test.

Logistic regression analysis showed that age, hemoglobin level, and HbA1C were independently associated with low HGS. Age above 75 years was associated with a high risk of low HGS, with an odds ratio of 20.47 (95% CI: 3.006–447.0, *p* = 0.011). High hemoglobin was inversely associated with low HGS, with an odds ratio of 0.526 for each unit increase (95%CI: 0.364–0.706, *p* < 0.001). High HbA1C levels were also associated with a high risk of low HGS, with an odds ratio of 1.324 for each unit increase (95%CI, 1.084–1.677, *p* = 0.011) (Table 4).

**Table 4:** Parameter estimates and odds ratio of factors associated with low HGS.

Variable	Estimate	Std. Err.	Z value	*p*-value	OR (95%CI)
Intercept	6.7107	1.9542	3.434	< 0.001	
Age > 75 years	3.0192	1.1861	2.545	0.011	20.47 (3.006–447.0)
Hb (per each unit increase)	−0.6431	0.1665	−3.863	< 0.001	0.526 (0.364–0.706)
HbA1c (per each unit increase)	0.2807	0.1100	2.553	0.011	1.324 (1.084–1.677)

### 3.4 ROC curve

**Figure 3:**
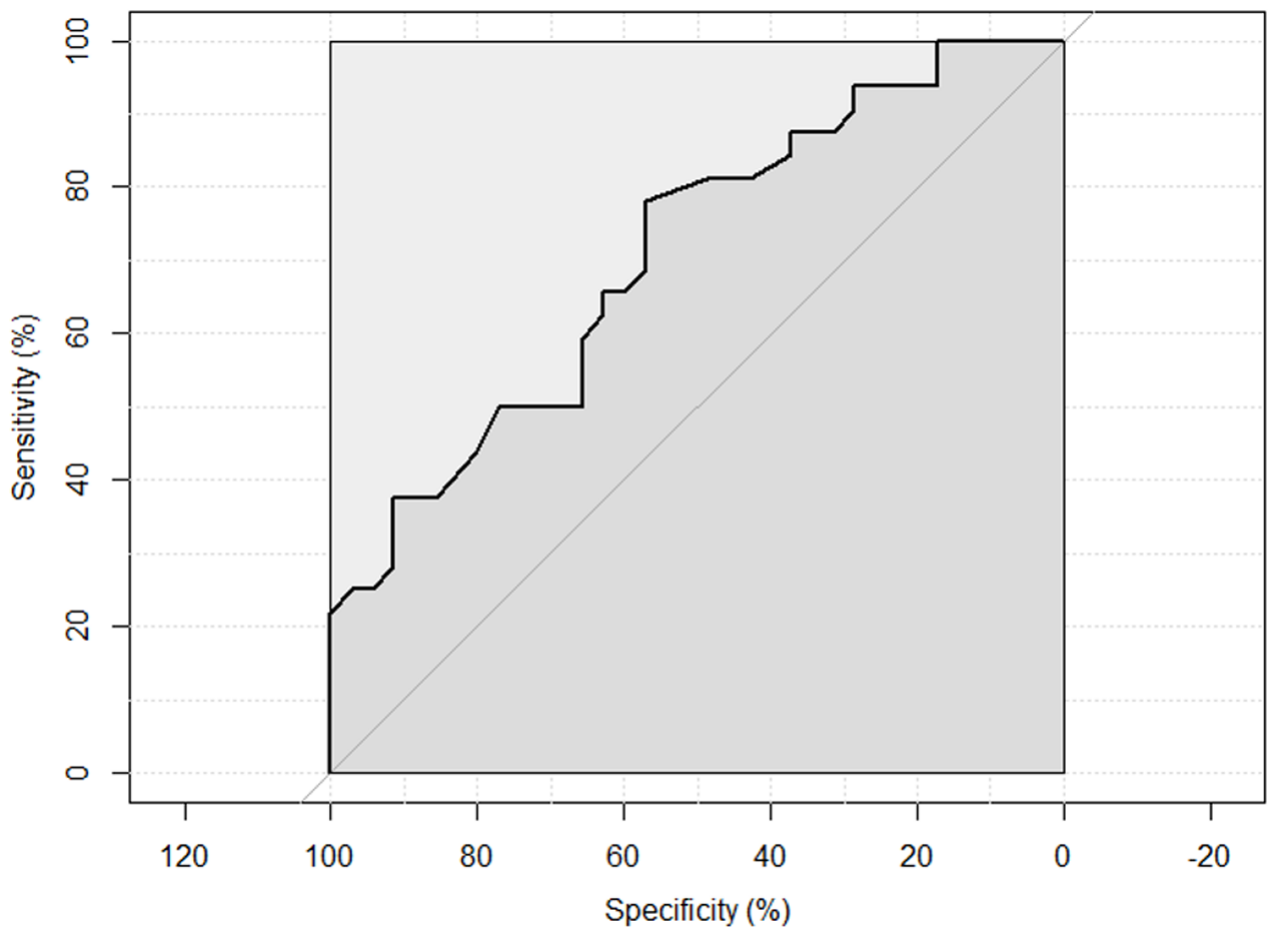
ROC curve of males to detect malnourished or at risk of malnourished using Hand Grip Strength Kg score.

**Figure 4:**
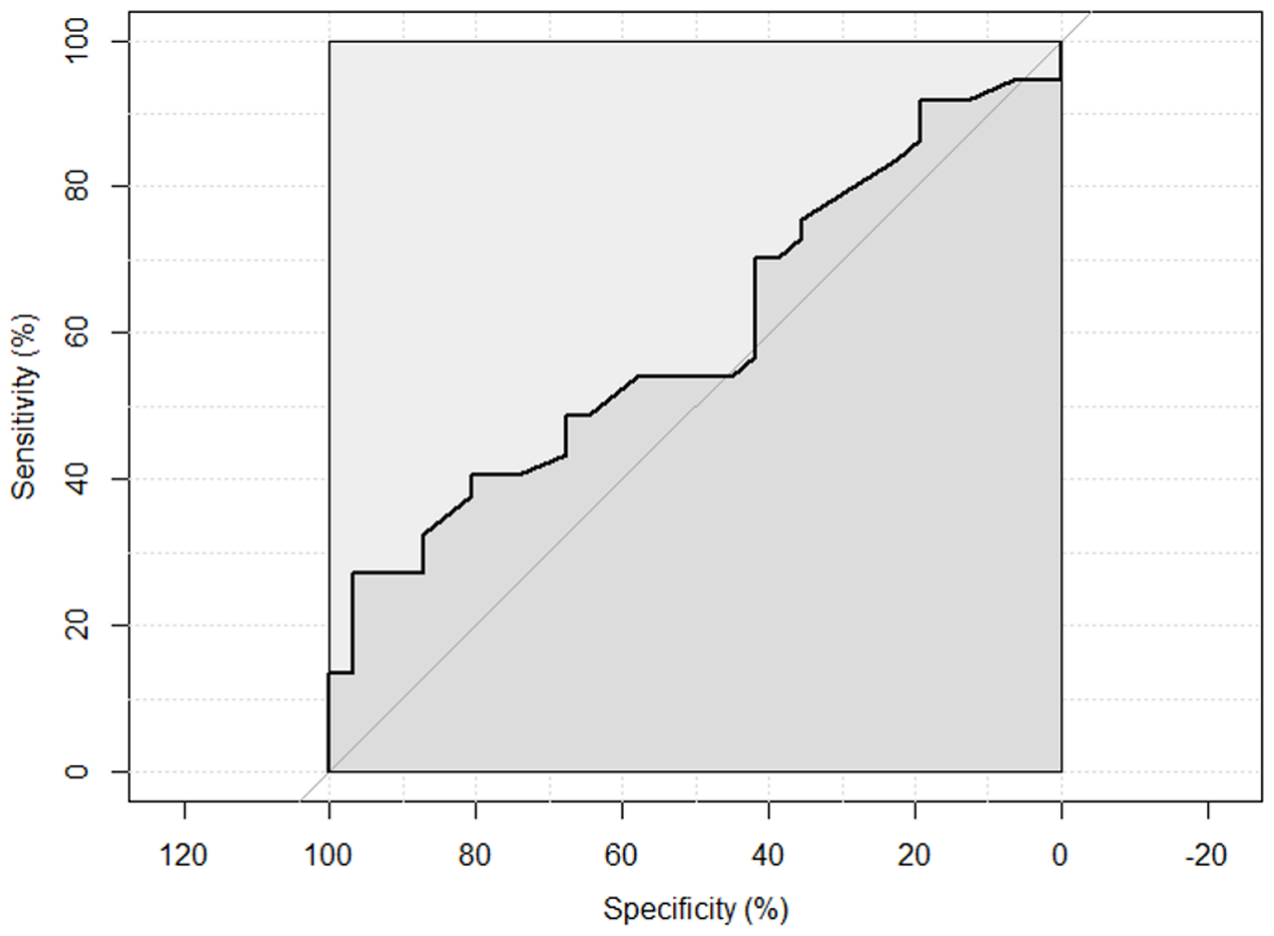
ROC curve of females to detect malnourished or at risk of malnourished using Hand Grip Strength Kg score.

ROC curve analysis was performed to examine the sensitivity and specificity of the observed HGS and nutritional status. An HGS () score of 27 for men showed a sensitivity of 93.75% and a specificity of 22.6% to detect malnourished or at risk of malnourished participants. ROC analysis showed an area under the curve of 70.8% (95%CI: 58.44–83.16%) (Figure 3). An HGS (kg) score of 16 for women showed a sensitivity of 86.5% and a specificity of 19.3% to detect malnourished or at risk of malnourished participants. ROC analysis showed an area under the curve of 59.9% (95%CI: 46.39–73.49%) (Figure 4).

**Figure 5:**
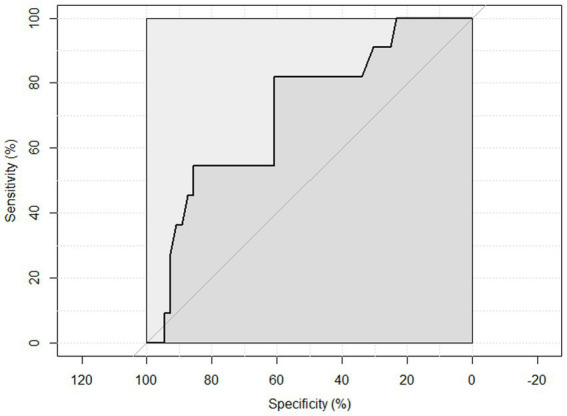
ROC curve of males to detect malnourished using Hand Grip Strength Kg score.

**Figure 6:**
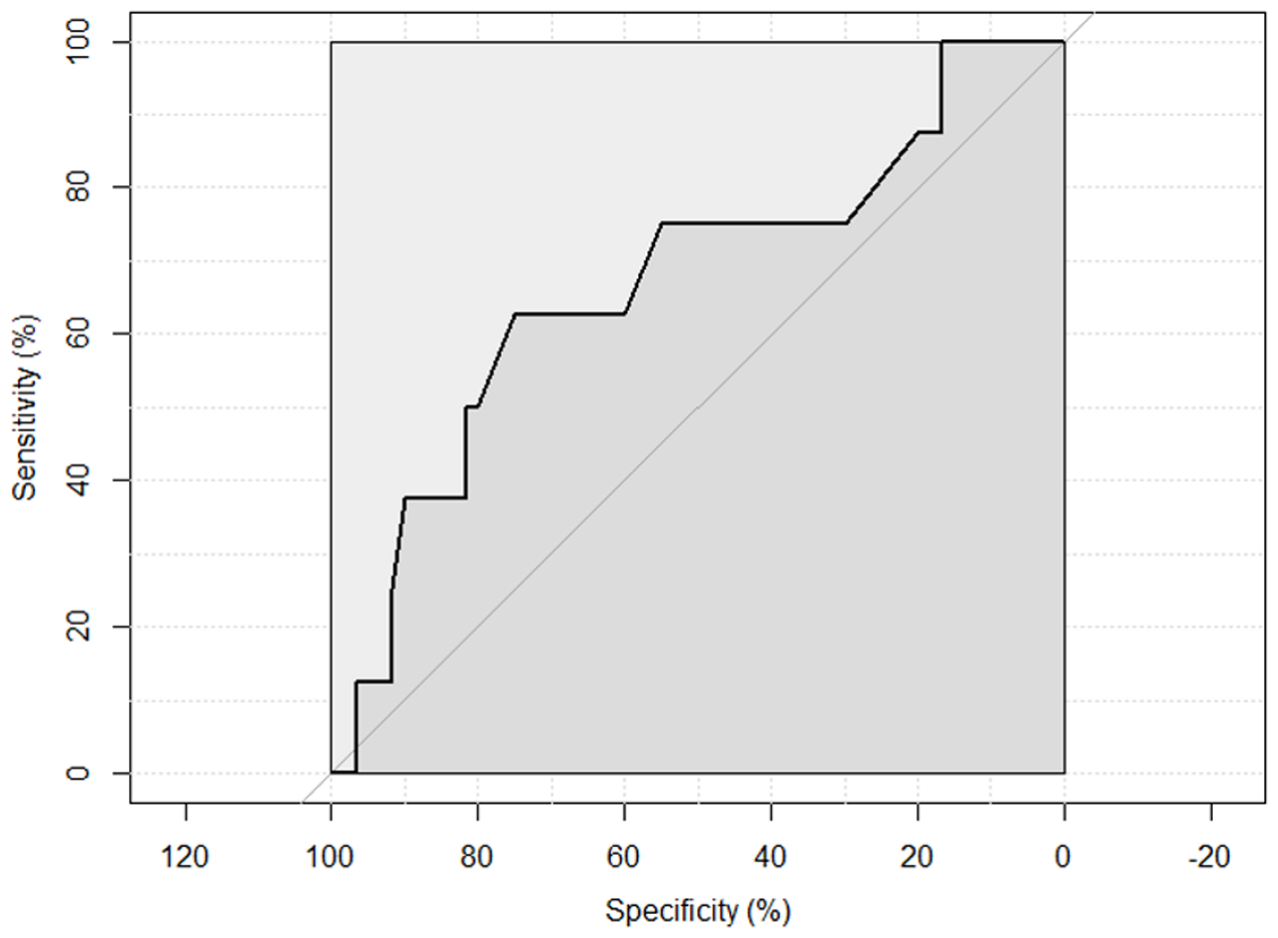
ROC curve of females to detect malnourished using Hand Grip Strength Kg score.

An HGS (kg) score of 27 for men showed a sensitivity of 100 and a specificity of 17.6% to detect malnourished participants. ROC analysis showed an area under the curve of 71.3% (95%CI: 58.64–88.06%) (Figure 5). An HGS (kg) score of 16 for women showed a sensitivity of 87.5% and a specificity of 16.7% to detect malnourished participants. ROC analysis showed an area under the curve of 67.2% (95%CI: 44.79–89.68%) (Figure 6).

## 4 Discussion

This study was conducted to estimate the correlation between HGS and nutritional status. Patients with low HGS on assessment had lower MNA-SF scores than those with normal HGS. This correlation has been confirmed in previous studies (12–14). Norman et al. also identified malnutrition as an independent risk factor for reduced muscle strength (7). Thus, there is a bidirectional relationship between HGS and nutritional status.

Our study identified lower HGS as a significant risk factor for both cardiac (*p* = 0.01) and kidney (*p* = 0.045) diseases. Low HGS has been found to increase the risk of cardiovascular mortality and cardiovascular events (15, 16). This link between HGS and cardiovascular outcomes can be attributed to a variety of possible explanations. People with a lower HGS may be generally less healthy than those with a higher HGS (17). Moreover, higher strength levels are often linked to engagement in resistance training, which, in turn, is associated with a reduced cardiovascular disease (CVD) risk score (18).

Our study also determined advancing age as an independent risk factor for low HGS in hospitalized older men and women (*p* = 0.006). This is in line with previous studies (19–21).

In addition, reduced HGS was strongly correlated with lower Hb levels (*p* < 0.05). According to Sutandyo et al. (21) and Gi et al. (22), a significant correlation between HGS and anemia was present in those over the age of 65 years. In the study by Hirani et al. (23), the decline in HGS in Australian older participants was directly related to the decline in Hb concentration. Pennix et al. (24) also reported that older participants with anemia had much lower HGS than those without anemia.

Furthermore, approximately 70% of our patients with low HGS had diabetes. Consequently, elevated levels of HbA1C were significantly correlated with low HGS. According to Mainous et al. (25) low HGS was associated with undiagnosed and diagnosed diabetes and hypertension, even among individuals with a healthy BMI. Similar findings were also reported by de Carvalho e Silva et al. (26), who identified incrementally lower grip strength in individuals with diabetes and osteoarthritis.

In our study, we observed a high sensitivity of HGS in detecting malnutrition among hospitalized older adults. However, its sensitivity was significantly low. This finding substantiates the hypothesis that HGS alone may not be sufficient for diagnosing malnutrition in this patient group.

The limited number of our participants diagnosed with malnutrition (*n* = 19) likely influenced this finding (19). Additionally, the cut-off points chosen for HGS measurement in this study may not have been optimized for our population and could have influenced the specificity of our results (19). White et al. (6) argued against the applicability of reduced HGS in cases of moderate malnutrition, highlighting potential limitations in its diagnostic utility. In addition, Norman et al. (7) suggested that in older individuals, muscle strength may serve as a marker of frailty rather than nutritional status. Moreover, factors such as the underlying causes of malnutrition and the duration of malnutrition could have influenced the variability in HGS measurements (19).

There were several limitations to our study. First, the relatively modest sample size could potentially reduce the generalizability of our findings. Second, the lack of consensus on HGS assessment protocols in the literature is likely associated with an increased probability of intra- and inter-test reliability. Third, the reliance on HGS cut-offs established by the European Working Group on Sarcopenia in Older People-2 (EWGSOP2), where low grip strength is defined as <16 kg for women and < 27 kg for men, may not be applicable to our population in Saudi Arabia and might have impacted the sensitivity and specificity results (27). Finally, several confounding factors, such as type of medications, socioeconomic status, and dietary habits, could influence both HGS and nutritional status, and these factors were not evaluated or analyzed in this study.

## 5 Conclusion

In our study, age above 75 years, low hemoglobin, and high HbA1C levels were independent risk factors for low HGS. Although low HGS showed high sensitivity in detecting malnourished individuals or those at risk of malnutrition, its specificity was low. Thus, relying solely on HGS may not suffice for assessing the nutritional status of hospitalized older adults. Replication of this study using locally reliable and validated HGS cut-off values for the Saudi population is warranted to confirm our findings.

## Data availability statement

The raw data supporting the conclusions of this article will be made available by the authors, without undue reservation.

## Ethics statement

The studies involving humans were approved by Research Ethics Committee (REC) of King Abdulaziz University. The studies were conducted in accordance with the local legislation and institutional requirements. The participants provided their written informed consent to participate in this study.

## Author contributions

SA: Writing – review & editing, Supervision, Project administration, Methodology, Formal analysis, Conceptualization. MS: Writing – original draft, Validation, Resources, Formal analysis, Data curation.
